# How Does Calcification Influence Plaque Vulnerability? Insights from Fatigue Analysis

**DOI:** 10.1155/2014/417324

**Published:** 2014-04-06

**Authors:** Baijian Wu, Xuan Pei, Zhi-Yong Li

**Affiliations:** ^1^Department of Engineering Mechanics, Southeast University, Nanjing 210096, China; ^2^School of Biological Science & Medical Engineering, Southeast University, Nanjing 210096, China; ^3^University Department of Radiology, University of Cambridge, Cambridge CB2 0QQ, UK

## Abstract

*Background.* Calcification is commonly believed to be associated with cardiovascular disease burden. But whether or not the calcifications have a negative effect on plaque vulnerability is still under debate. *Methods and Results.* Fatigue rupture analysis and the fatigue life were used to evaluate the rupture risk. An idealized baseline model containing no calcification was first built. Based on the baseline model, we investigated the influence of calcification on rupture path and fatigue life by adding a circular calcification and changing its location within the fibrous cap area. Results show that 84.0% of calcified cases increase the fatigue life up to 11.4%. For rupture paths 10*D* far from the calcification, the life change is negligible. Calcifications close to lumen increase more fatigue life than those close to the lipid pool. Also, calcifications in the middle area of fibrous cap increase more fatigue life than those in the shoulder area. *Conclusion.* Calcifications may play a positive role in the plaque stability. The influence of the calcification only exists in a local area. Calcifications close to lumen may be influenced more than those close to lipid pool. And calcifications in the middle area of fibrous cap are seemly influenced more than those in the shoulder area.

## 1. Introduction

Rupture of atherosclerotic plaque is a major cause of human mortality worldwide, which makes the prerupture identification of vulnerable atheroma extremely important for patient risk evaluation. Evidences have shown that the composition of an atherosclerotic plaque, rather than its degree of stenosis or size, is usually of more importance for acute clinical events. Generally a vulnerable plaque is often found to be associated with a thin fibrous cap, a high inflammation burden, a large lipid pool, macroscopic heterogeneity, and so on [1, 2]. Calcification is commonly believed to be associated with cardiovascular disease burden [3–7]. Recently, the influence of calcification on plaque vulnerability has raised many research interests [8–12]. There are many ways to image the calcification in plaque, such as noninvasive molecular imaging probes. Chen and Dilsizian [13] used the molecular probe 18 F-sodium fluoride (18 F-NaF) for positron emission tomography (PET) imaging, which targets active microcalcifications in atherosclerotic plaques. Kimura et al. [14] revealed a significantly higher frequency of lipid-rich plaque with microcalcification in lesions with echo signal attenuation.

The role that the calcification plays in plaque vulnerability is still under debate. Some studies indicated beneficial effects in stabilizing the plaque, making it stiffer and less prone to rupture [15, 16], while others tended to believe it would increase the risk of plaque rupture [3, 17]. Studies from Mauriello et al. [18] showed that the calcification, as well as its distance from the lumen, is not correlated with the presence of unstable plaques. Thus, the authors suggested that the calcification is not useful to identify the vulnerable plaque. Hermann et al. [19] found that individuals suffering a stroke have significantly higher coronary artery calcification (CAC) values at baseline than the remaining individuals, and furthermore CAC is an independent stroke predictor in addition to classical risk factors for those patients at low or intermediate vascular risk. Moreover, mechanical experiments on human carotid plaques by Mulvihill et al. [20] showed that calcification in the tissue structure may lead to increased vulnerability of the plaque. On the other hand, it was demonstrated by Shaalan et al. [21] that symptomatic plaques are less calcified and more inflamed than asymptomatic plaques, implying that the calcification may reduce the plaque rupture risk. Wahlgren et al. [22] investigated thirty carotid endarterectomy plaques which were classified as noncalcified and calcified and obtained a similar result that fibrous cap inflammation is more likely to occur in noncalcified than in calcified plaques, suggesting that plaque calcification may result in protection against the rupture of plaque.

Computational studies on microcalcifications have also been investigated previously. Kelly-Arnold et al. [23] examined the spatial distribution, clustering, and the shape of different microcalcification size in fibrous caps and found that nearly all fibrous caps have microcalcifications, but only a small subset has rupture potential. Bluestein et al. [24] developed a fluid-structure interaction (FSI) model to study the microcalcification effects on the plaque vulnerability and found that calcification can increase plaque vulnerability. Cilla et al. [25] investigated the effect of microcalcifications on the stress field of an atheroma plaque vessel section by performing a parametric finite element study on an idealized model. Vengrenyuk et al. [26] investigated the stress distribution using the multilevel micro-CT based 3D numerical modeling techniques. Results showed that the peak circumferential stress increases with the existence of calcifications (inclusions) and may grow even higher by elongated microcalcifications, while in contrast, macrocalcifications in cap shoulders were shown to actually increase the plaque stability.

Despite the above viewpoints that the stress induced by normal blood pressure or shearing flow characterizes the vulnerability of plaques, another possible mechanism that the rupture may result from fatigue accumulating process has been investigated [27–29]. The remaining fatigue life of plaque thus may be used to evaluate the rupture risk [30, 31]. In the current study, we investigated the influence of calcium deposition on plaque rupture from the fatigue crack growth point of view. Here, we built an idealized model in which only one calcification is included. Based on the model, we investigated the influence of calcification on crack path and fatigue life. Moreover, we changed the calcification location in order to inquire its impact.

## 2. Methods

An idealized model was created with a blunt crescent-shaped lipid pool and a circular calcification embedded. The cross-section includes 5 parts: the arterial wall, the fibrous cap, the lumen, the lipid pool, and the calcified inclusion. The baseline cross-section without the calcification had the thickness of the fibrous cap as 10 mm, the thickness of the lipid pool as 15 mm, and the angle of the lipid pool which is used to control the length of the lipid pool as 45°.

**Figure 1:**
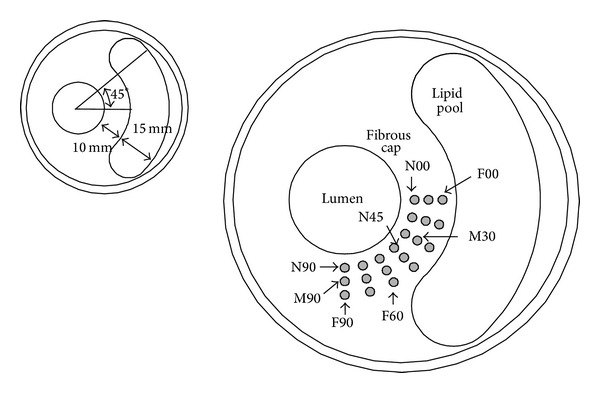
Different calcification cases. Here the capitals N, M, and F, respectively, mean a near, middle, and far distance from the lumen; and the number after the letter means the angle from the *x*-axis.

The calcification was assumed to be a circular inclusion in fibrous cap area with the radius *r* of 0.8 mm. Calcified inclusions are put into variable locations to investigate their influence of the fatigue life as well as the vulnerability of the plaque. Here two parameters, the distance from the lumen *d* and the angle from the *x*-axis *α*, were used to locate the calcification center. In the study, *d* varied among 2.5 mm (Near), 5.0 mm (Middle), and 7.5 mm (Far), and *α* was among 0, 15°, 30°, 45°, 60°, 75°, and 90°. Totally 21 different calcification locations as shown together in Figure 1 were investigated. In Figure 1, the capital letters N, M, and F, respectively, mean a near, middle, and far distance from the lumen; and number next to the letter means the angle from the *x*-axis.

In our study, plaque rupture was understood as a result of fatigue process under cyclic blood pressure. As the stress at the crack tip is infinity, we adopt stress intensity factor (SIF) *K* to describe the status at the crack tip. The Paris Law was used to calculate the crack growth rate. Paris found that the fatigue crack growth rate is related to the change of SIF within one stress cycle, and the equation is

(1) $$\begin{array}{c} \frac{\mathrm{d}a}{\mathrm{d}N}=C\cdot \Delta K^{m}. \end{array}$$

Here *a* is the crack length. *N* is the number of cycles, namely, the number of heartbeats. *K* is the SIF change within one stress cycle (one heartbeat), namely, the *K* under the systolic pressure minus that under diastolic pressure. *C* and *m* are material constants. Since no fatigue test on human plaque has been reported so far, here the outcome from rubber will be used and *C* and *m* are, respectively, chosen given as 3.16E-5 and 2.12 [32]. Using a different value of *C* and *m* would not change the general conclusions for this study.

The maximum circumferential stress criteria [33] were adopted to calculate the crack growth direction. In the theory, the growth angle *θ*, defined as the angle between the growth path and the local *x'*-axis, is determined by

(2) $$\begin{array}{c} \theta =\arctan\frac{K_{II}}{\sqrt{K_{I}^{2}+8K_{II}^{2}}}-\arctan\frac{3K_{II}}{K_{I}}. \end{array}$$

In numerical simulation, the initial crack should be created first. Then the finite element model of the cracked vessel is solved. With the obtained Δ*K*(*a*_*j*_), the crack growth rate and direction can be calculated through (1) and (2), respectively. The new crack tip could thus be predicted. And with updated crack tip, the above calculation starts again. This loop keeps running until the crack reaches the boundary of plaque boundary, that is, the lipid pool or artery wall. At this time, we will judge the plaque as “*ruptured*,” and the crack growth path **L** as well as the total number of heartbeats *N*_*r*_ could be obtained. Thus, the plaque life *T* for this rupture path **L** could be estimated as

(3) $$\begin{array}{c} T_{L}=\frac{N_{\text{r}}}{\text{Heart}\;\text{rate}}. \end{array}$$

As said above, the study includes a baseline vessel and 21 calcified vessels. Here for each* vessel case*, we also introduce many different crack initial locations. Each crack initialization—we called it one* computational case*—leads to a crack growth path **L** and its corresponding plaque life *T*_**L**_. These crack growth paths could be understood as possible rupture paths for the plaque. Thus, the value of computed plaque life could thus be used to evaluate the possibility of a rupture path. A longer life implies a lower rupture risk within a fixed and upcoming time period, one year for instance, and vice versa.

In the study, initial cracks in baseline cross-section were manually created from 0° to 180° with a step of 9°, respectively (21 cases). Correspondingly, the results are used as baseline values for comparison. Initial cracks for calcified cross-sections are created mainly from *α* − 45° to *α* + 45°, also with a step of 9° (10~12 cases for each cross-section). This is because the calcification is relatively in a very small size that cracks initialized farther than *α* ± 45° will lead to nearly the same results as that of the baseline.

The numerical simulation was implemented in the finite element software ABAQUS (Version 6.10, Providence, RI). The distributed blood pressure (a systolic pressure of 120 mm Hg and a diastolic pressure of 80 mm Hg) is applied on the lumen surface. The heart rate is given as 70 per minute here. 8-node quadrilateral elements are used to mesh all models, while collapsed triangle elements are distributed around the crack tip.

Linear and elastic constitutive relation is used for all plaque components. The Young modulus of the arterial wall, the fibrous cap, the lipid pool, and the calcification was chosen as *E*_*w*_ = 0.3 Mpa, *E*_*p*_ = 0.6 Mpa, *E*_*l*_ = 0.02 Mpa, and *E*_*c*_ = 10 Mpa, respectively [30]. Poisson's ratio for each component was set as *ν*_*w*_ = *ν*_*p*_ = *ν*_*l*_ = *ν*_*c*_ = 0.48.

## 3. Results

**Figure 2:**
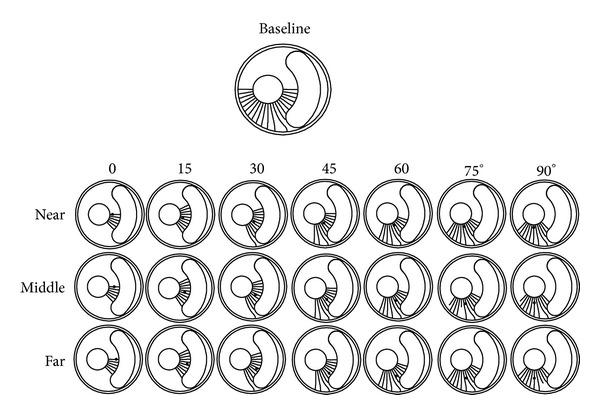
Rupture paths for all cases.

As said above, our computations covered the baseline vessel and 21 calcified vessels. Also, there are 21 crack attempts of initialization (computational cases) for the baseline and 10~12 for the calcified cases. Totally 21 + 231 = 252 cases are calculated. Results are shown in Figure 2, in which a rupture path corresponds to a computational case. It could be found that the calcification seemly will not significantly change the baseline rupture directions.

The fatigue life changes due to the calcification at all crack paths are investigated, aiming to answer the following three questions. (1) Does the calcification influence the plaque vulnerability, positively or negatively? (2) How large a scope of area the calcification will influence? (3) Does the influence depend on the location of calcification?

### 3.1. Calcification May Increase Plaque Stability

**Figure 3:**
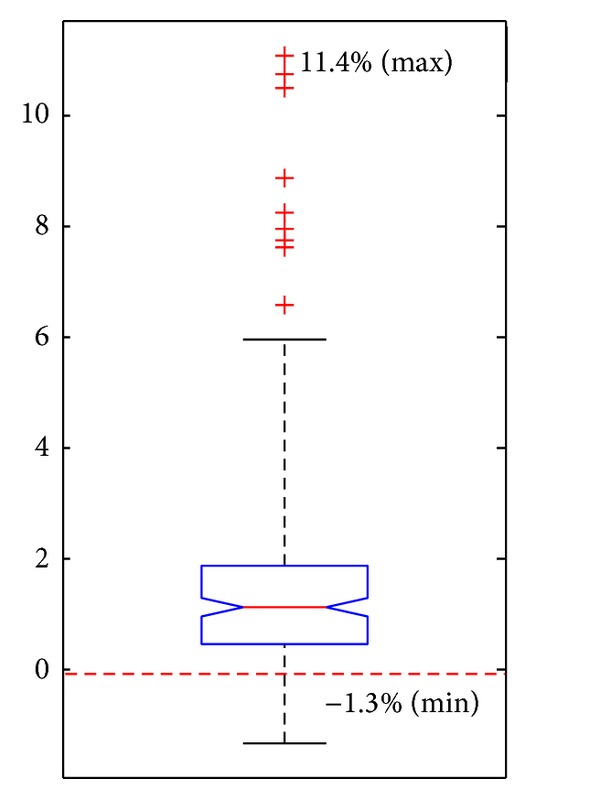
Fatigue life changes (%).

All data are collected and box-plotted together in Figure 3. It could be seen that, for 194 out of 231 calcified cases (84.0%), the fatigue life of the plaque increases up to 11.4%. In contrast, the biggest life decrease is just −1.3%, which actually could be ignored. Therefore, it could be inferred that the existence of calcifications may play a positive role in the plaque stability.

### 3.2. Influence Scope

**Figure 4:**
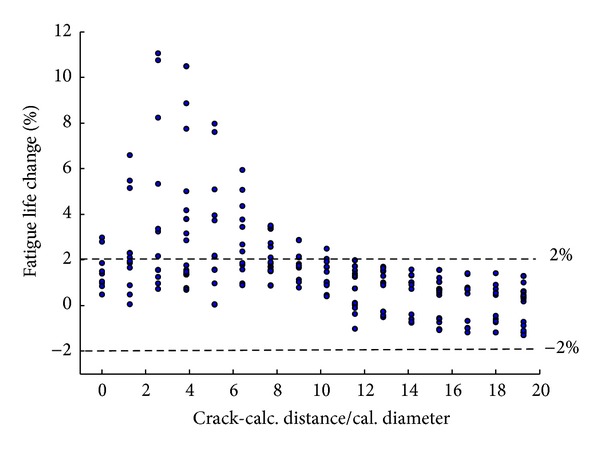
Relationship between crack-calcification distance and fatigue life changes.

To investigate scope of area in which the calcification may influence, all results are regrouped and plotted together in Figure 4. Here the *x*-axis is a ratio between crack-calcification distance *d*_CL_ and the calcification diameter *D* (1.6 mm), where *d*_CL_ means the distance from the calcification center to the calculated rupture path. The *y*-axis is the fatigue life changed (in percentage) due to the calcification.

It could be found that the calcification most possibly influence the rupture paths of which the *d*_CL_/*D* are among 1~6, while for those paths directly going through the calcification, fatigue life seemly changes little. Also, it is found that when *d*_CL_/*D* > 10, the influences are all below ±2%. It is said that, for areas 10*D* far from the calcification, the influence is negligible. The influence scope of area is about 10*D*.

### 3.3. Calcification Locations

**Figure 5:**
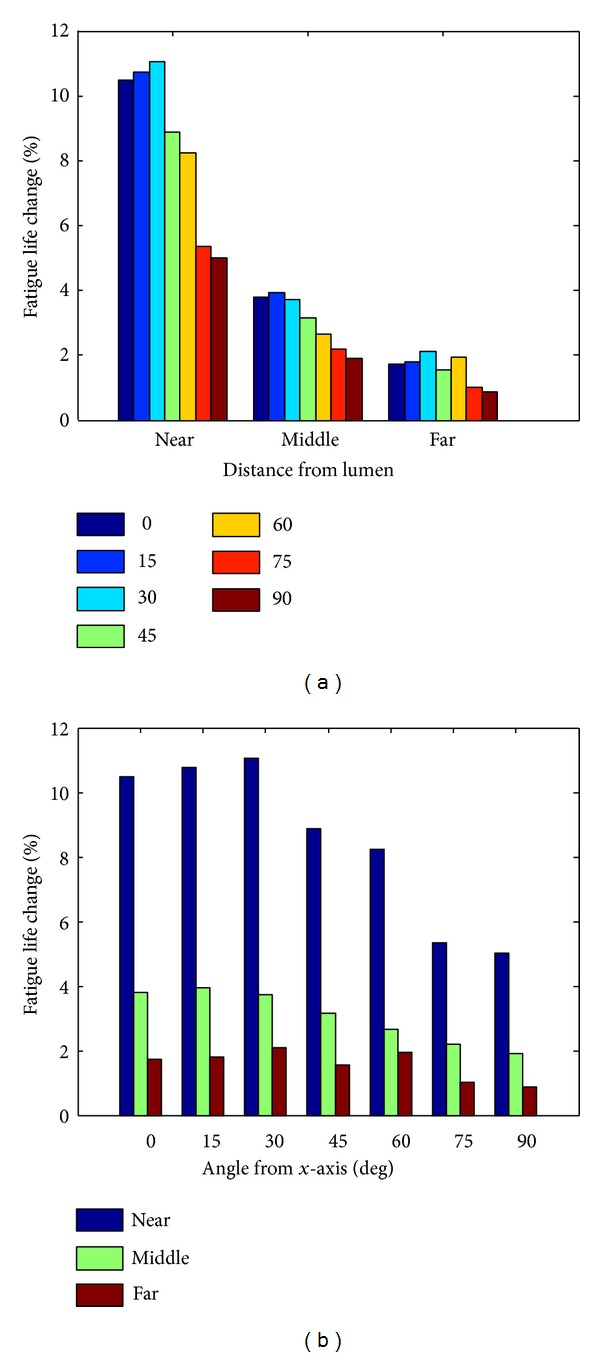
Calcification locations and fatigue life changes.

To investigate if the above influence of calcifications depends on their locations, first for each calcified cross-section, we extract the max life changes among all crack paths. Then results for all 15 calcified vessels are plotted together in Figure 5. It is found that calcification close to lumen may be influenced more than those close to lipid pool. Besides that, calcifications locating in the middle area (small *α*) of fibrous cap are seemly influenced more than those in the shoulder area (large *α*).

## 4. Discussion

**Figure 6:**
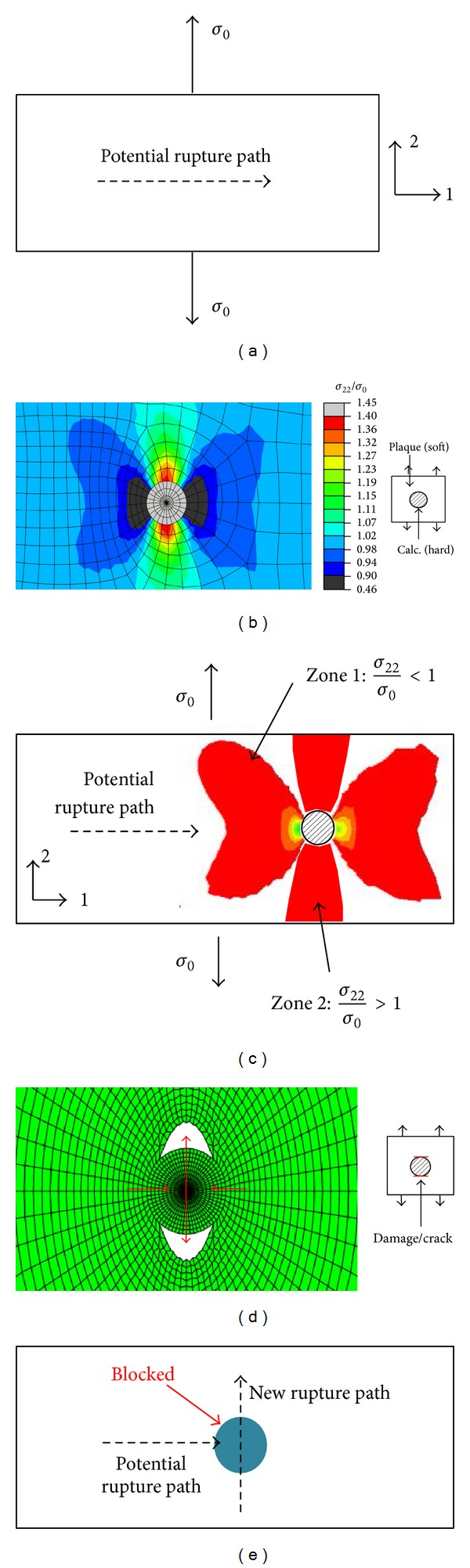
Diagram of the possible mechanism of reducing rupture risk.

The role that the calcification plays in plaque vulnerability is controversial. In the study, the results tend to show that it may increase plaque stability, which generally agrees with the viewpoints in [21, 22, 34]. Here we try to explain the mechanisms from the biomechanical insights. First, as we know, the rupture is mainly due to the tensile force/stress. If the potential rupture path is known, locally the dominated stress status should be in tension and be perpendicular to the rupture path (Figure 6(a)). We define a local coordinate system here that 1-direction and 2-direction are parallel to the rupture path and the tensile stress, respectively. Then the calcification, a hard inclusion embedded in a soft matrix (the plaque), is considered.  Figure 6(b) shows the result of *σ*_22_ field induced by the calcification and the remote tensile stress. It is found that, in the neighborhood of the calcification, stress in Zone 1 (Figure 6(c)) slightly decreases and in Zone 2, the stress increases; that is to say, the risk of plaque broken in Zone 1 decreases, while the broken risk in Zone 2 increases. Now, we assume a crack or plaque damage occurs on the upper and lower side (the largest stress concentration location as shown in Figure 6(b)), for which the deformation field could be solved and shown in Figure 6(d). It could be found that still due to the hard inclusion (the calcification) the left and right sides extrude the calcification even more, and at the same time, the damage/crack in the upper and lower side intensifies. A new rupture direction in fact generates and blocks the direction of original rupture path (Figure 6(e)), which may finally explain why the calcification increases the stability.

From the classical viewpoint that the maximal stress value characterizes the vulnerability, here the plaque stability should decrease because the stress near the upper and lower side of the calcification significantly increases, as shown in Figure 6(b). It should be noted that researches have shown that ruptures often (for about 40%) occur in regions where the numerical model does not predict the maximal stress [8]. Here we suggest that detailed analysis on failure modes/paths should also be taken into account. For example, obviously the stress concentration happens in nearly all material heterogeneous cases. However, equally as a heterogeneous inclusion, a hard calcification or a weakened void caused by inflammation may lead to totally different results even though they all have stress concentrations around.

It is interesting that the calcification influences the rupture paths a little far from it (with *d*_CL_/*D* among 1~6) more than those directly going through it. This may coincide with the mechanisms revealed above that, for cases of 1 < *d*_CL_/*D* < 6, cracks may grow into Zone 2 and retard due to the reason shown in Figure 6(e). Seemly for cases *d*_CL_/*D* < 1, the retard of crack growth which is mainly caused by the stress reduction in Zone 1 is not so significant.

The position of calcification is thought to be of importance for rupture risk stratification by some researches [35, 36], where it is found that calcification locating in middle cap area is influenced more than that in shoulder area. Results are similar in this study (Figure 5). As for the distance of calcification from the lumen, this study shows that calcification close to lumen may be influenced more than that close to lipid pool, while some studies reported that they have no obvious difference [18]. In our study, areas near to lumen usually have greater circumferential tensile stresses than those far from lumen. Greater stresses usually lead to a more rapid crack growth, which consequently will change the life more. This may be the reason for our outcome.

It should be noted that only one calcified inclusion embedded in fibrous cap is considered. Actually there may have several calcifications coalesced together, which may lead to extra influences. Also, the baseline fibrous cap is not an extremely thin case; a much thinner cap possibly has other effects because the calcification may strongly influence the stress distribution nearby. These two aspects are planned to be considered.

## 5. Conclusion

Calcifications may play a positive role in the plaque vulnerability—at least not negative. In our study, the max fatigue life increase is about 12% with the radius of calcification 0.8 mm. Considering that the practical size is much less, actual influence may be even small. The influence of the calcification is local rather than global, which is mainly concentrated in an area in the size of 10*D* neighborhood. It is found that calcification close to the lumen may be influenced more than those close to the lipid pool. And calcifications located in the middle area of fibrous cap are seemly influenced more than those in the shoulder area. In all, since calcification would not increase the rupture risk, ignoring the calcification is acceptable. At least it will not lead to an underestimated risk.
